# Analysis of Meridian Flow Direction by Electrical Stimulation Method

**DOI:** 10.1186/s11671-022-03694-4

**Published:** 2022-07-08

**Authors:** Yan-Wen Liu, Chuan-Wei Kuo, Ting-Chang Chang, Yu-Chiang Hung, Yung-Fang Tan, Chia-Chuan Wu, Chien-Hung Lin, Wen-Chung Chen, Wen-Long Hu, Tsung-Ming Tsai

**Affiliations:** 1 Department of Chinese Medicine, Kaohsiung Chang Gung Memorial Hospital, 123 Dapi Road, Kaohsiung, 83301 Taiwan; 2 Department of Materials and Optoelectronic Science, National Sun Yat-Sen University, 70 Lienhai Rd., Kaohsiung, 80424 Taiwan; 3 Department of Physics, National Sun Yat-Sen University, 70 Lien-hai Road, Kaohsiung, 80424 Taiwan; 4 The Center of Crystal Research, National Sun Yat-Sen University, 70 Lienhai Rd., Kaohsiung, 80424 Taiwan; 5 School of Chinese Medicine, Chang Gung University College of Medicine, 259, Wenhua 1st Rd., Guishan District, Taoyuan, 33302 Taiwan; 6 Department of Electrical Engineering, National Sun Yat-Sen University, 70 Lienhai Rd., Kaohsiung, 80424 Taiwan; 7 Kaohsiung Medical University of Medicine, 100, Shiquan 1st Rd., Kaohsiung, 80708 Taiwan; 8 Fooyin University College of Nursing, 151, Jinxue Rd., Kaohsiung, 83102 Taiwan

**Keywords:** Traditional Chinese medicine (TCM), Electrical acupuncture, Meridian directionality, Drift current

## Abstract

Meridians constitute the theoretical foundation of acupuncture in traditional Chinese medicine (TCM), and they have been described for 2000 years. Classical TCM advocates for the directionality of meridians. Finding an accurate method to verify this directionality is an important goal of TCM doctors and researchers. In this study, we objectively explored the physical properties of meridians, such as response current from electrical stimulation, to explore their directionality. The Agilent B1500A semiconductor measurement analyzer was utilized to input the alternating current waveforms and detect the response current on the meridians. The results showed that the direction of the meridians influences the intensity of the response current. Therefore, the mechanisms behind the directions of ion transportation and the meridians were investigated using the response time and the intensity of the response current. Thereafter, we propose a model to explain this mechanism. Afterward, a comparison between the direction of the meridian in this experiment and ancient Chinese medicine classics was performed.

## Introduction

Since 1979, the World Health Organization (WHO) has published indications of acupuncture, which has become a commonly accepted method for treatment. As complementary alternative medicine, acupuncture has been applied for decades, and it has been established to be therapeutically effective by modern researches [1]. Regardless of the acupuncture method, such as needle insertion, manual pressure, electrical stimulation, magnets, and low-power lasers, the theoretical bases are meridians. Meridians provide an important foundation for traditional Chinese medicine (TCM) and guidelines for clinical practice. The “meridian theory" was described in the Yellow Emperor's Classic of Internal Medicine for 2000 years [2]. Finding an accurate method to verify the existence of meridians is an important goal for TCM doctors and researchers [3–8].

For example, many anatomical structures have been proposed to describe meridians, such as tendinomuscular meridians or primo vascular systems (or Bonghan Ducts) [9, 10]. Various studies have revealed the different parts of the meridian. Gerhard Litscher’s group found that infrared thermography cannot visualize stimulation-induced meridian-like structures [11]. Wei et al*.* constructed a meridian acupoint temperature map by reporting the characteristics of the meridian acupoint temperatures in healthy medical students [12].

In addition, the Ryodoraku theory explores the utilization of electric conductivity to identify meridians. The Ryodoraku theory was proposed by Dr. Yoshio Nakatani in the 1950s. This theory states that there are some points on the skin that have a high electric conductivity, and the energy change of these points could represent the condition of the body, i.e., the inner viscera or the balance of qi and blood. These points are called Ryodoten, and studies have shown that they connect to form a line, which is called Ryodoraku. The location of the Ryodoraku is almost identical to the location of the meridian distribution. Consequently, the energy of a Ryodoraku is considered to represent the energy of the corresponding meridian [13, 14].

The meridian energy analysis device (MEAD) is an instrument developed based on the Ryodoraku theory [15]. MEAD detects the electrical conductivity of the skin over meridian lines. It is believed that the electrical conductivity of the meridian reflects the biological energy or balance of qi and blood in the body [15]. Due to the therapeutic effects of some treatments, such as analgesic response or control of blood pressure, acupuncture is thought to be related to the central or peripheral nervous system [16, 17]. Thus, in this research, the existence of the meridian was fully confirmed [18].

However, the Ryodoraku theory only detects the electrical conductivity of the skin over a single acupoint of the meridian. Meanwhile, the anatomical or morphological pieces of evidence on the existence of the meridian are still lacking, and the exploration of the meridian with modern physical devices has been minimally investigated. A previous study was referenced [19], and the characteristics of the current–time (I–t) curve measuring body meridians are similar to those obtained by the isothermal transient ionic current (ITIC) theory. Based on the drift and diffusion currents of the theory, the method of conducting electricity in the meridians is mainly through ions. Electrical stimulation was used to determine the electrical characteristics of the meridian, which serves as a precedent for meridian research in the field of physical electronics.

Based on the intrinsic properties of meridians, in this study, we investigated the mechanism of the direction of ion transport and transmission of the meridians mentioned above. The effectiveness of the meridian direction is studied utilizing electrical characteristics, and the results are compared to those in TCM. The meridian direction is based on ancient Chinese medicine classics. It is generally believed that the course of the three Yin meridians of the hand is from the thorax to the hand; the course of the three Yang meridians of the hand is from the hand to the head; the course of the three Yang meridians of the foot is from the head to the foot; and the course of the three Yin meridians of the foot is from the foot to the thorax [20, 21]. In this study, a semiconductor measurement analyzer (Agilent B1500A) was utilized to input the waveforms with different voltage polarities on the three Yin meridians of the hand (Taiyin lung meridian of the hand, Jueyin pericardium meridian of the hand, and Shaoyin heart meridian of the hand) and the three Yang meridians of the foot (Yangming stomach meridian of the foot, Shaoyang gallbladder meridian of the foot, and Taiyang bladder meridian of the foot). Two acupoints were chosen over each meridian, and the differences between the acupoints on each meridian were recorded. Finally, the correlation between the experimental results and the meridian direction recorded in the TCM classics was shown. This study provides the basis for meridian directionality for further research and clinical treatment choices.

## Experimental Methods

### Participant Selection

The Institutional Review Board of the Chang Gung Medical Foundation approved this clinical trial (IRB permit no. 201901601A3). Before enrollment, all participants received informed consent forms, which they completed signed after understanding the project. Thereafter, the experiments commenced. Thirty volunteers who met the inclusion criteria of being healthy and above 18 years were recruited (15 men and 15 women). Pregnant and lactating women, people who were fasting, people with idiopathic thrombocytopenic purpura (ITP), and people who take sleeping pills or psychiatric drugs were excluded. No specific acupoints were targeted; instead, the acupoints were chosen from convenient locations which are favorable for acupuncture.

### Inclusion and Exclusion Criteria

According to a prospective survey, acupuncture can have some side effects despite its benefits [22]. As an invasive treatment, it may sometimes induce local or systemic adverse reactions [22–24]. Moreover, a systemic review showed that life-threatening events may also develop, albeit rarely. Consequently, the exclusion criteria were stringent. Bleeding and hematoma are the most common adverse reactions; therefore, volunteers with bleeding tendencies (platelet count of less than 20,000 and/or thrombocytopenic purpura) were excluded. Volunteers with chronic medical conditions, who were prescribed anticoagulants, were also excluded. Pregnant women and volunteers with pacemakers were excluded. No adverse reaction was observed throughout the experiment.

### Measurement Machine and Equipment

In this study, all electrical signals were obtained using an Agilent B1500A semiconductor parameter analyzer and a Cascade M150 microprobe station. The acupuncture needles used were produced under the same conditions, in the same factory (Dong Bang Acupuncture Inc.), and on the same day to minimize experimental error.

### Experiment Process

**Fig. 1:**
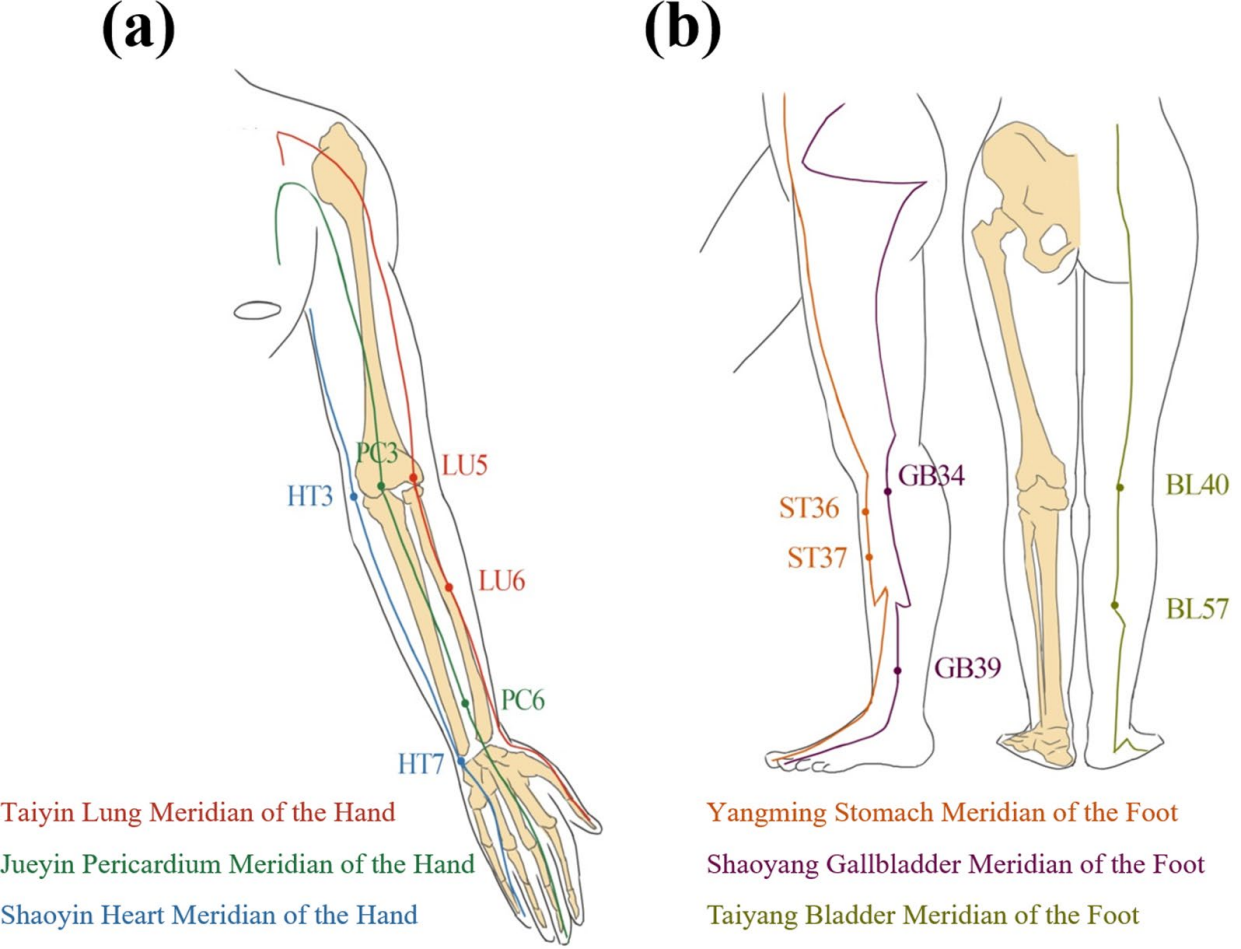
Selected acupoints: **a** Yin meridians of the hand and **b** Yang meridians of the foot

The decision of the left- or right hand and the left- or right foot provided by each subject to participate in this experiment was categorized as random. We chose two acupoints over each Yin meridian of the hand: LU5 (Chize) and LU6 (Kongzui) from the Taiyin lung meridian of the hand, PC3 (Quze) and PC6 (Neiguan) from the Jueyin pericardium meridian of the hand, and HT3 (Shaohai) and HT7 (Shenmen) from the Shaoyin heart meridian of the hand; two acupoints over each Yang meridian of the foot: ST36 (Zusanli) and ST37 (Shangjuxu) from the Yangming stomach meridian of the foot, GB34 (Yanglingquan) and GB39 (Xuanzhong) from the Shaoyang gallbladder meridian of the foot, and BL40 (Weizhong) and BL57 (Chengshan) from the Taiyang bladder meridian of the foot. The detailed locations are featured in Fig. 1 and Table 1.

**Table 1:** Selected acupoints with detailed locations

Acupoints	Location
*Taiyin Lung Meridian of the Hand*	
LU5(Chize)	Located in the transverse cubital crease, on the radial side (laternal side) of the tendon of musculus biceps branchii
Lu6(Kongzui)	On the line between LU5(Chize) and LU9 (Tasiyuang), 7 cm above the transverse crease of the twist
*Jueyin Pericardium Meridian of the Hand*	
PC3 (Quze)	Located in the transverse cubital crease, on the ulnar side (medial side) of the tendon of musculus biceps branchii
PC6 (Neiguan)	Between the tendons of m. palmaris longus and m. flexor carpi radialis, 2 cun above the transverse crease of the twist
*Shaoyin Heart Meridian of the Hand*	
HT3 (Shaohai)	At the middle part of the line between the medial end of the transverse cubital crease and medial epicondyle of the humerus when the elbow is flexed. Flex the elbow to locate the point
HT7 (Shenmen)	At the ulnar end of the transverse crease of the wrist, on the radial side of the tendon of m. flexor carpi ulnaris
*Yangming Stomach Meridian of the Foot*	
ST36 (Zusanli)	3 cun below Dubi (ST35), one finger width laternal to the anterior crest of the tibia
ST37(Shangjuxu)	3 cun below ST35 one finger width laternal from the anterior crest of the tibia
*Shaoyang Gallbladder Meridian of the Foot*	
GB34 (Yanglingquan)	In a depression anterior and inferior to the head of the fibula
GB39 (Xuanzhong)	3 cun above the tip of the laternal malleolus, in the depression of the anterior border of the fibula
*Taiyang Bladder Meridian of the Foot*	
BL40 (Weizhong)	Midpoint of the transverse cubital of the popliteal fossa between the biceps femoris and semitendinosus tendons
BL57 (Chengshan)	In the depression below the gastrocnemius muscle, 8 cun inferior to BL40

**Fig. 2:**
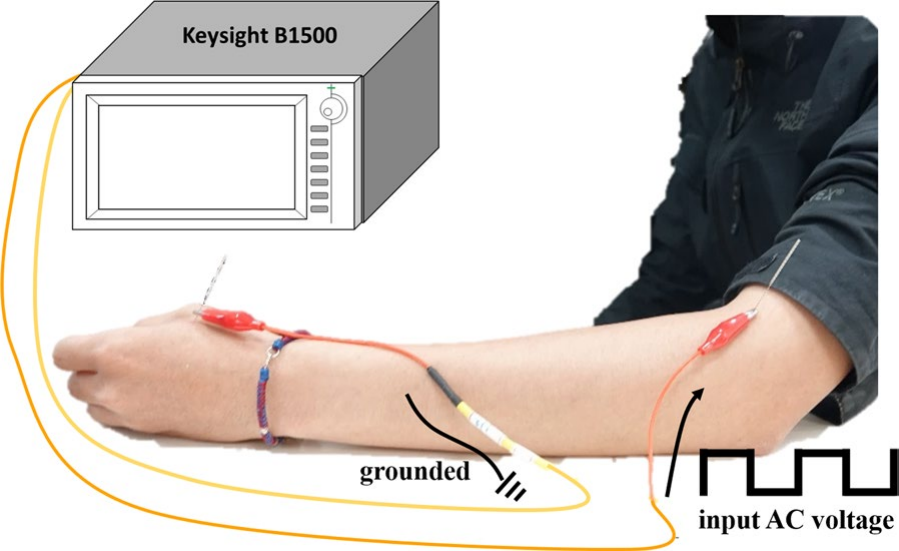
Schematic of the electrical acupuncture

After fully explaining the experimental procedures, the consent forms were signed, and acupuncture was performed by a certified TCM doctor. Disposable 1 cun (C.M.S. acupuncture needles, 0.30 × 25 mm) and 1.5 cun sterilized acupuncture needles were employed for the upper and lower limbs, respectively. All acupoints were sterilized using 75% alcohol. The locations of the acupoints were detected based on clinical experience and ancient classics. After the acupuncture needles were inserted, acupuncture manipulation was performed to achieve deqi-soreness, numbness, or distension, as described by participants, and heaviness, tension, tightness, or fullness, as described by the acupuncturist [9, 25]. After deqi, alligator clips were used to clip the acupuncture needles. Meanwhile, the Agilent B1500A parameter analyzer inputted the alternating current (AC) waveforms on two acupoints. In this study, the AC waveform considered was a continuous square wave oscillating between 2 and − 2 V with a frequency of 2 Hz. The first acupoint was near the heart (called the near heart point (NHP)), while the second was far from the heart (called the far heart point (FHP)). Conversely, the analyzer received the response current, *I*_response_, from the grounded FHP/NHP. A schematic of the experiment is shown in Fig. 2.

**Fig. 3:**
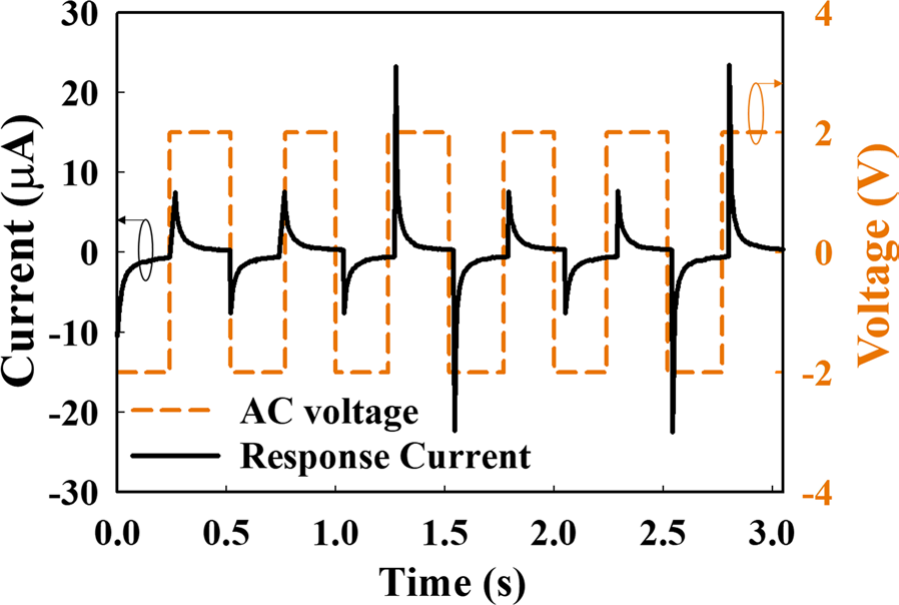
The relationship between the input wave and *I*_response_

The order of the upper limb acupoints is as follows: the Taiyin lung meridian of the hand, Jueyin pericardium meridian of the hand, and Shaoyin heart meridian of the hand. After the completion of the hand experiments, the foot experiments were performed in the following order: the Yangming stomach meridian of the foot, Shaoyang gallbladder meridian of the foot, and Taiyang gallbladder meridian of the foot. The relationship between the input wave and *I*_response_ is shown in Fig. 3.

## Result and Discussion

**Fig. 4:**
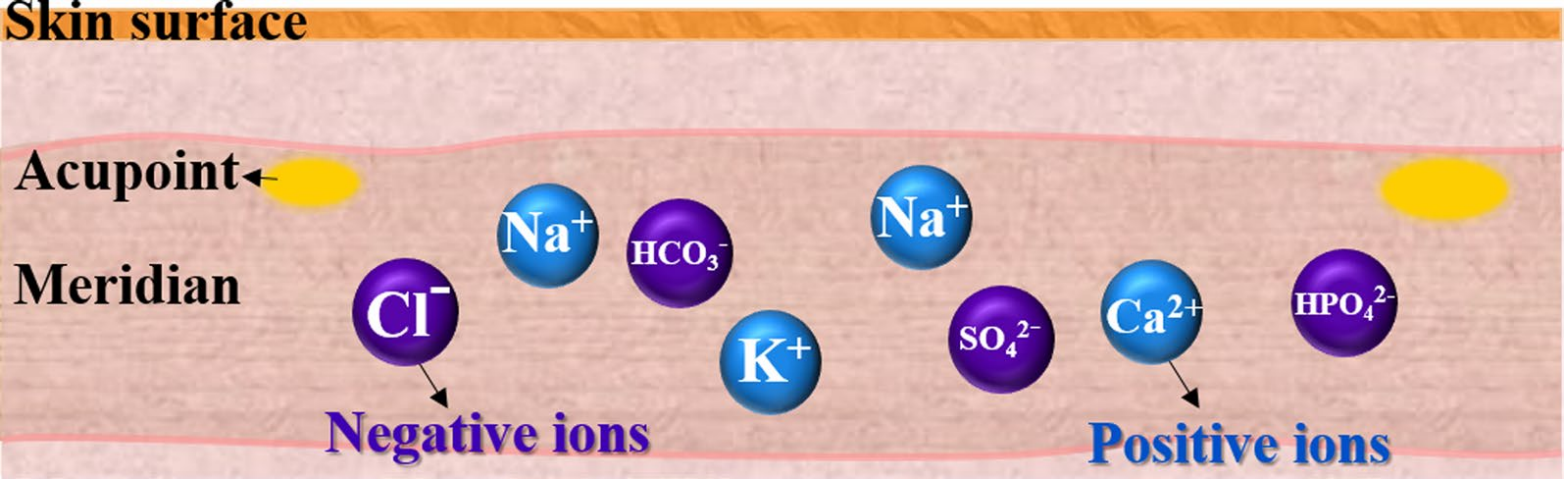
Uniform distribution and diffusion of ions in the meridian without the electron field

An external force, such as an electric field, is utilized to determine the properties of the current, such as direction and intensity, which is a conventional technique in semiconductor studies. In this study, the direction of the meridians was identified using the external electric field. Electrical acupuncture was utilized to control the flow of ions, which produced current; thereafter, the current was enhanced or countervailed by the force of the meridian. The intensity of the current increased when the direction of the meridians and the extra current forward flow. The described current is defined as the direction of positive ions. Before the electrical signal input, the ions in the meridian were uniformly distributed and underwent diffusion, as shown in Fig. 4. When an AC wave was inputted on an acupoint using the parameter analyzer, the ions drifted along the direction of the electric field, which is called the drift current (*I*_drift_). When the drifting ions create an ion concentration gradient due to the electric field, a diffusion current is also generated. Hung et al. reported that the response current of ion flux includes ion drift and diffusion currents with an applied square AC wave [19]. However, the contribution of the diffusion current to the response current can be ignored. Thus, herein, only the effect of drift current is discussed.

When a negative voltage was inputted on the FHP, positive ions were attracted to the FHP (along the electric field direction) and negative ions were repelled to the NHP (opposite the electric field direction). Meanwhile, the received *I*_response_ is higher and lower since the direction of the meridian is identical and opposite direction, respectively.

**Fig. 5:**
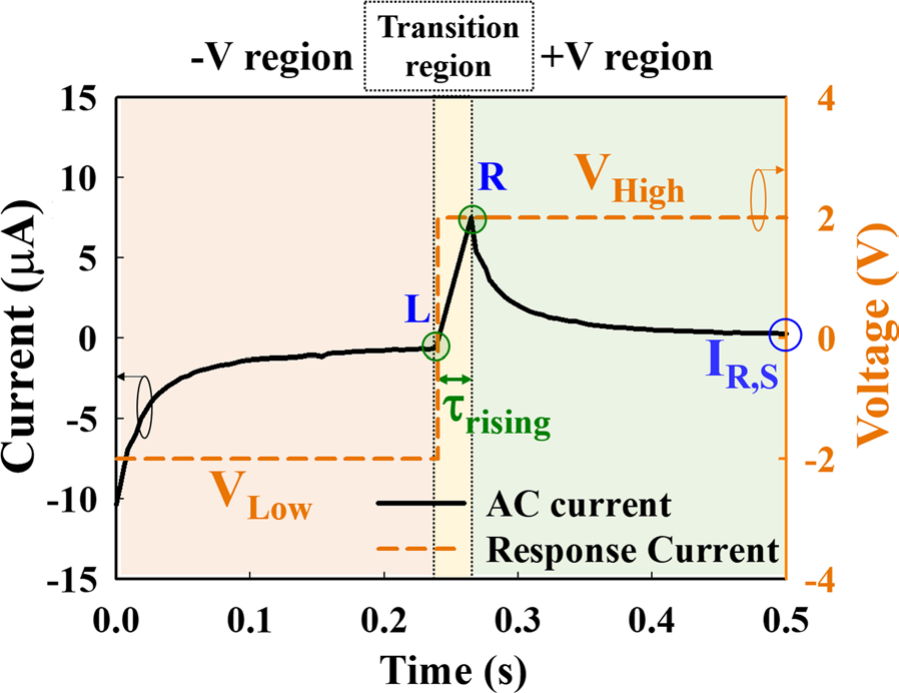
Time evolution of *I*_response_ and the input voltage. *V*_Low_ and *V*_High_ represent the negative and positive voltages of the input AC voltage, respectively

Based on the applied electric field and the mechanism of the drift current [26], the *I*_response_ shown in Fig. 5, one period of *I*_response_ can be divided into three regions according to the change in current with increasing time. At the beginning of the I–t measurement, the current increases gradually in the − V region when a negative voltage (*V*_Low_) is inputted. Thereafter, *I*_response_ reaches point L, and the current increases dramatically in the transition region (point L to point R). The *τ*_rising_ parameter represents the time from the negative saturation of *I*_response_ to the positive maximum of *I*_response_, which responds to the input wave switch from a negative voltage to a positive voltage (*V*_High_). Subsequently, the current decreases gradually and approaches a flat condition in the + V region.

**Fig. 6:**
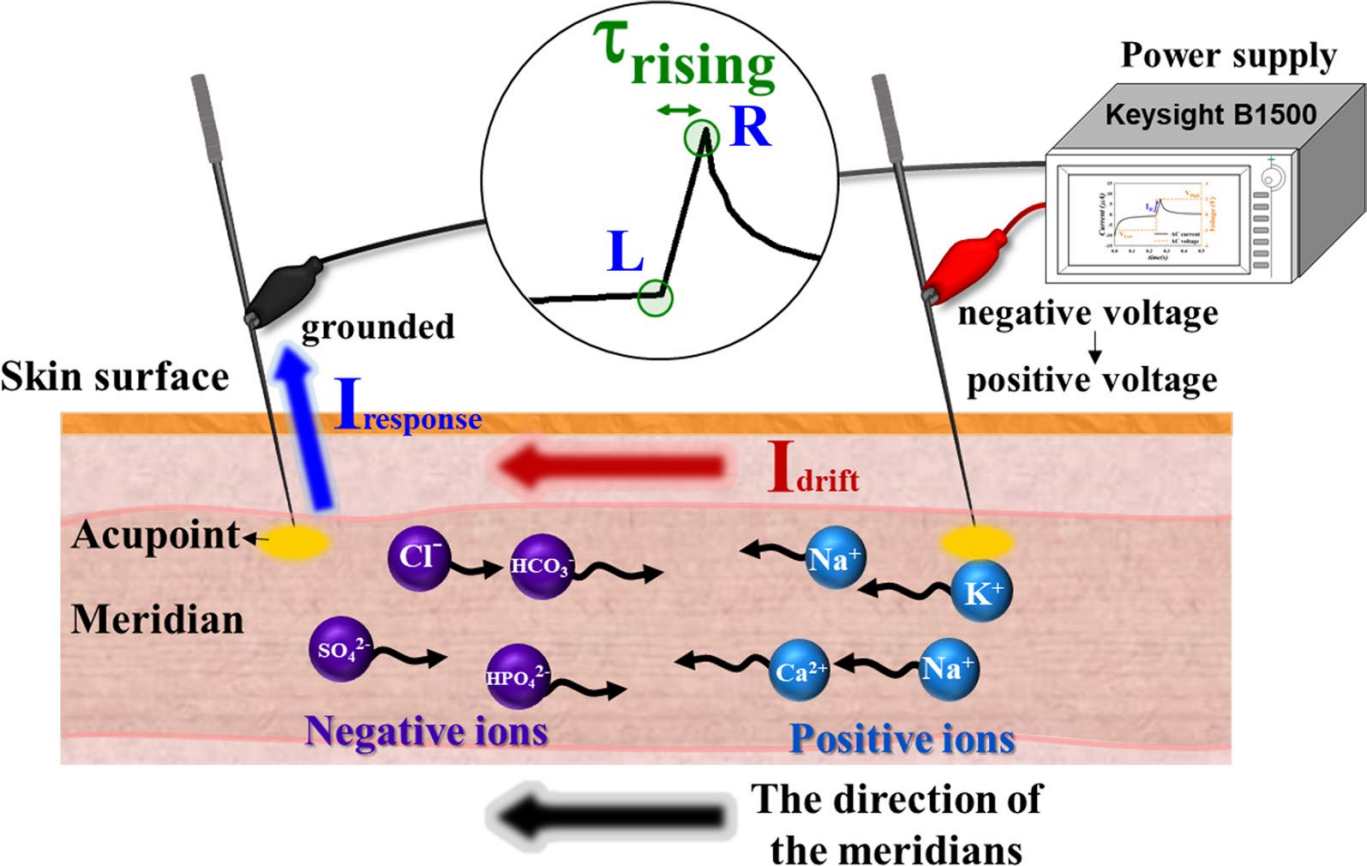
The direction of the positive and negative ions when the inputted negative voltage changes to a positive voltage of AC wave. Many positive and negative ions exchange instantaneously

**Fig. 7:**
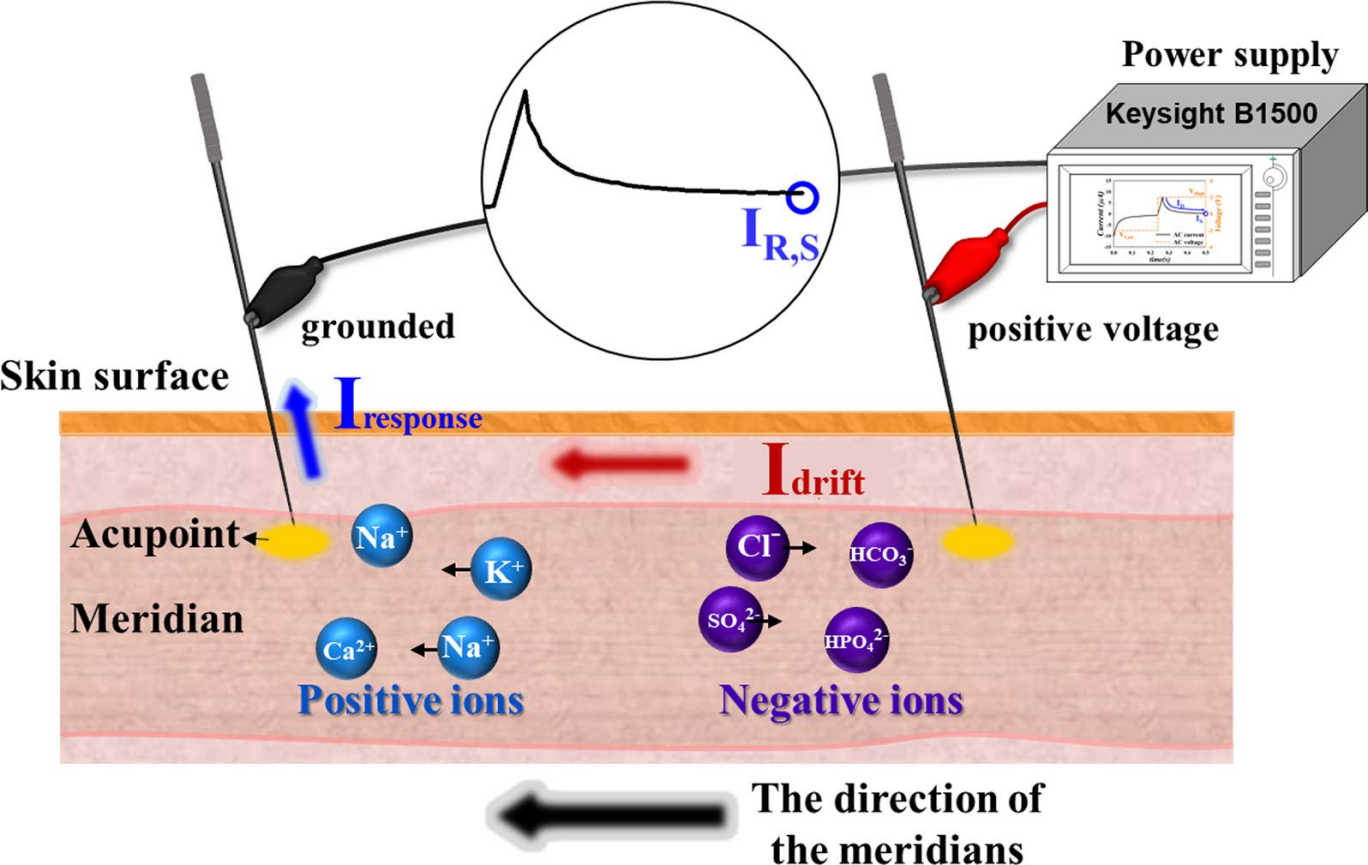
The direction of the positive and negative ions after applying positive voltage for a long time. The ions accumulate at the acupoint

In the − V region, because a negative voltage was applied, the positive ions drifted along the electric field direction and removed the grounded acupoint; thus, the *I*_response_ value was negative. Therefore, with increasing time, ions accumulate near the acupoints (negative ions near the grounded acupoint and positive ions near the input acupoint), causing the received *I*_response_ to approach a flat. In the transition region, the input voltage changes from a negative to a positive value in an ultrashort time, causing a large number of ions to exchange suddenly, as shown in Fig. 6. Finally, with increasing the input time of the positive voltage in the + V region, most ions tend to accumulate near the acupoints, and the parameter analyzer receives a decreased and saturated *I*_response_ (I_R,S_), as shown in Fig. 7. The peak of *I*_response_ was received with different peak high when a voltage (AC square waveform) was input to the acupoint. To reduce the variation factor in the experiment, the comparison of *I*_response_ was used at the saturation point.

**Fig. 8:**
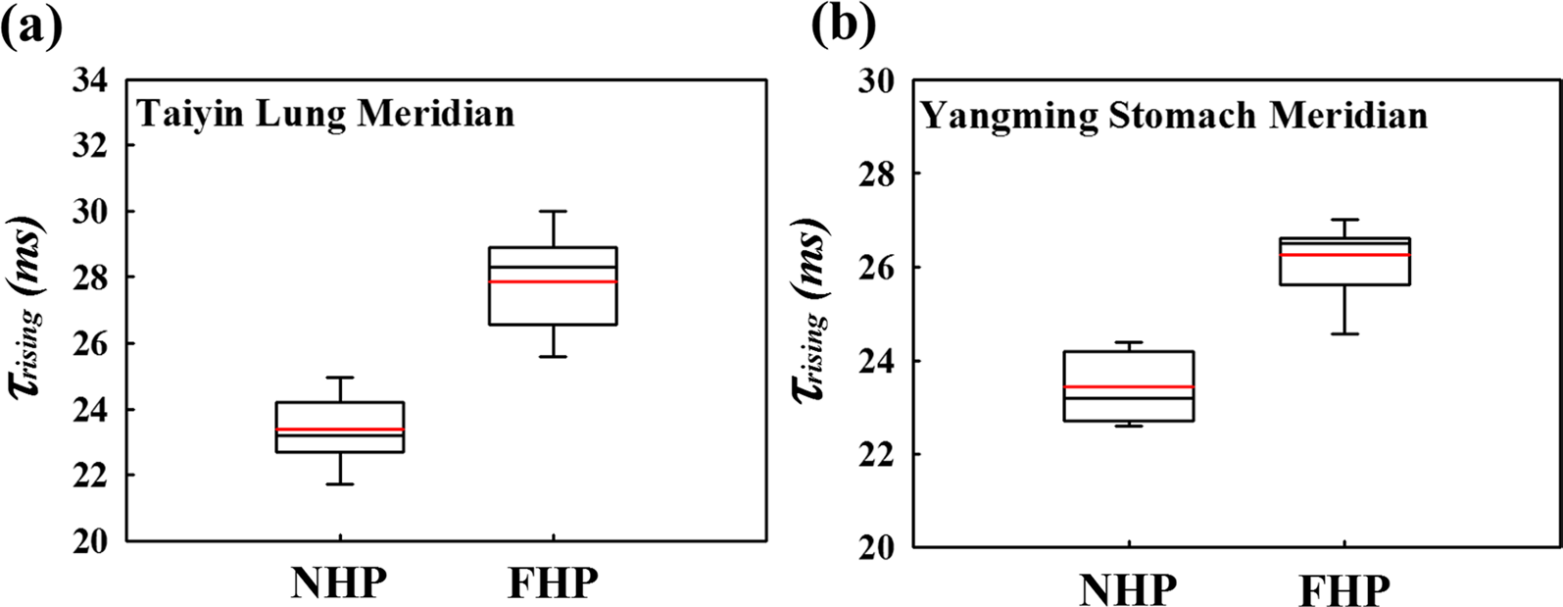
Box plot of *τ*_rising_ of the **a** Taiyin lung meridian and **b** Yangming stomach meridian. The red line in the box plot is the mean value of the data

**Fig. 9:**
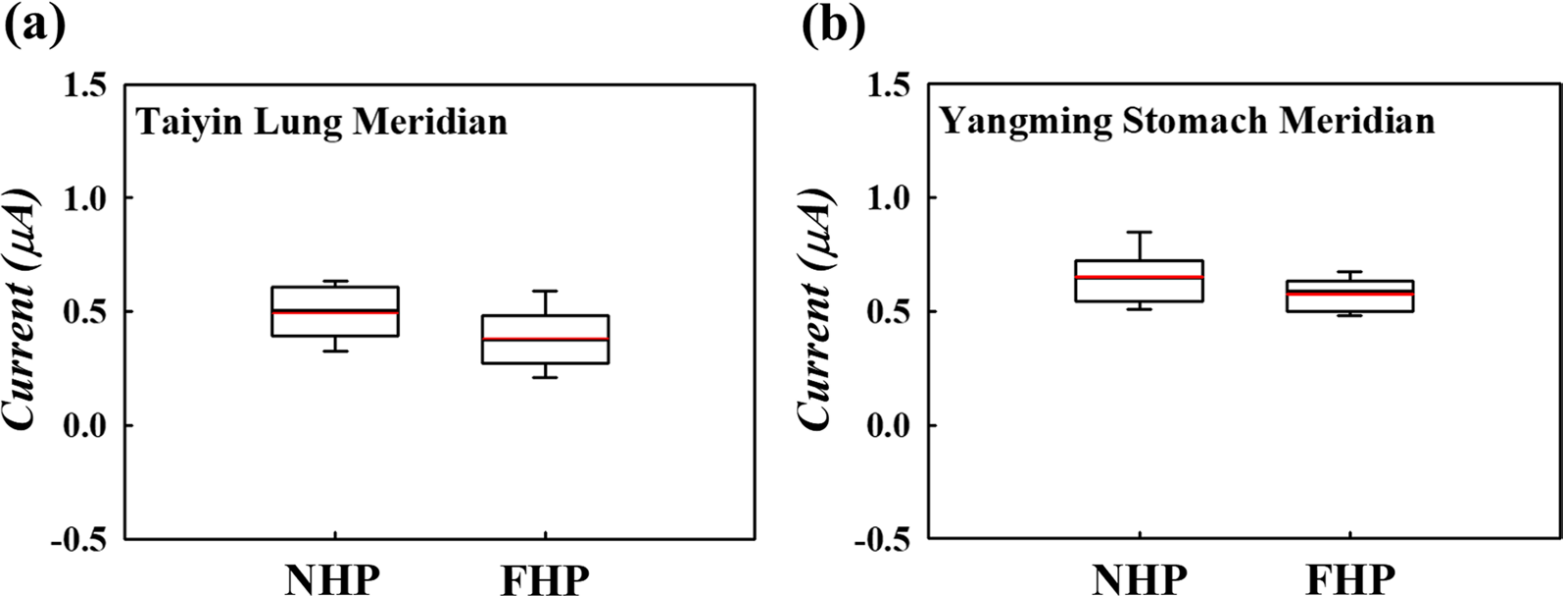
Box plot of the mean current of the **a** Taiyin lung meridian and **b** Yangming stomach meridian. The red line in the box plot is the mean value of the data

To analyze the relationship between the meridian direction and the I_response_, the *τ*_rising_ and *I*_*R,S*_ were examined, and the differences between the meridians were compared. The average *τ*_rising_ of the Taiyin lung meridian of the hand in Fig. 8a is 23.3 ms when the wave input is on NHP and 28.8 ms when wave input is on FHP. The average *τ*_rising_ of the Yangming stomach meridian of the foot in Fig. 8b is 23.3 ms when the wave input is on NHP and 26.5 ms when the wave input is on FHP. According to Fig. 8, the average *τ*_rising_ with an input wave on the NHP is less than that on the FHP. Contrary to the above *τ*_rising_ results, the average *I*_*R,S*_ with an input wave on the NHP is higher than that on the FHP, as shown in Fig. 9. The average *I*_*R,S*_ value of the Taiyin lung meridian of the hand in Fig. 9a is 0.49 μs when the wave input is on NHP and 0.38 μs when the wave input is on FHP. The average *I*_*R,S*_ of the Yangming stomach meridian of the foot in Fig. 9b is 0.65 μs when the wave input is on NHP and 0.57 μs when wave input is on FHP. The *τ*_rising_ and *I*_*R,S*_ values of the Taiyin lung meridian of the hand, Jueyin pericardium meridian of the hand, Shaoyin heart meridian of the hand, Yangming stomach meridian of the foot, Shaoyang gallbladder meridian of the foot, and Taiyang bladder meridian of the foot are shown in Tables 2 and 3, respectively. According to Tables 2 and 3, when the wave input is applied from NHP to FHP, the *τ*_rising_ is lower and *I*_*R,S*_ is higher of six meridians. The box plots show that the statistics of the meridian data in this study are concentrated around the central tendency and without extreme values. All data evaluations in this study were repeated three times and are presented as the mean ± standard deviation.

**Table 2:** The arithmetic means and standard deviation of the *τ*_rising_ values of the Sanyang meridian of the hand and Sanyin meridian of the foot

Raising time (ms)	NHP	FHP	*P* value
*Meridians*			
Taiyin Lund Meridian	23.3 ± 0.8	28.8 ± 2.7	8.7e − 08
Jueyin Pericardium Meridian	24.2 ± 1.5	29.0 ± 1.9	2.5e − 05
Shaoyin Heart Meridian	24.5 ± 1.7	30.9 ± 3.2	1.3e − 05
Yangming Stomach Meridian	23.3 ± 0.8	26.5 ± 0.1	1.4e − 10
Shaoyang Gallbladder Meridian	23.6 ± 1.1	27.0 ± 1.0	3.6e − 05
Taiyang Bladder Meridian	23.8 ± 1.7	28.5 ± 2.2	1.9e − 05

**Table 3:** The arithmetic means and standard deviation of the *I*_R,S_ of the Sanyang meridian of the hand and the Sanyin meridian of the foot

Mean Current (μA)	NHP	FHP	*P* value
*Meridians*			
Taiyin Lund Meridian	0.49 ± 0.11	0.38 ± 0.13	1.3e − 05
Jueyin Pericardium Meridian	0.74 ± 0.27	0.62 ± 0.21	2.8e − 05
Shaoyin Heart Meridian	0.23 ± 0.18	0.21 ± 0.12	7.7e − 05
Yangming Stomach Meridian	0.65 ± 0.13	0.57 ± 0.07	1.1e − 05
Shaoyang Gallbladder Meridian	0.77 ± 0.14	0.63 ± 0.19	8.5e − 05
Taiyang Bladder Meridian	0.80 ± 0.25	0.70 ± 0.12	4.5e − 05

**Fig. 10:**
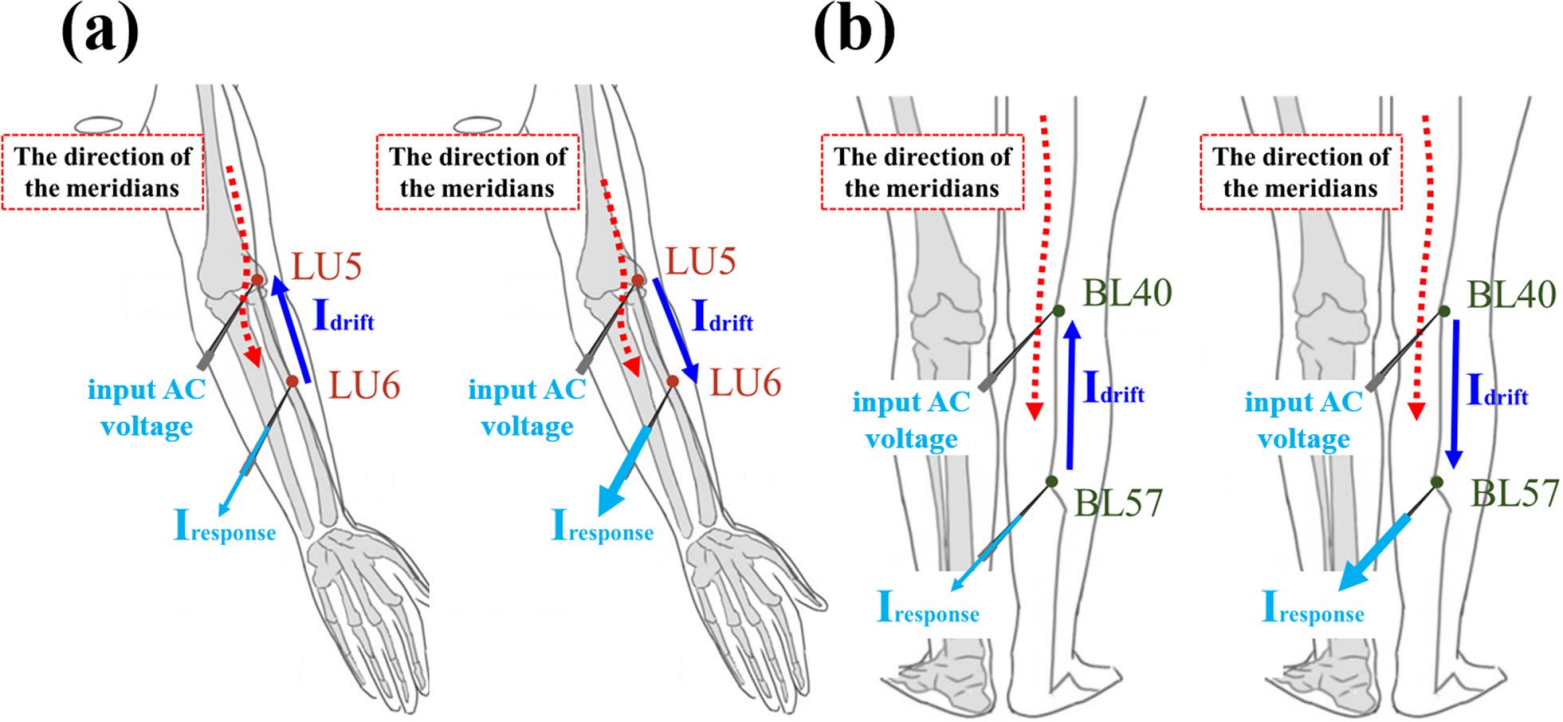
Relationship between the direction of *I*_drift_ and the direction of the meridian of **a** the hand and **b** the foot. The reverse and the forward relationships between the *I*_drift_ and the meridian direction caused a decrease and an increase in the *I*_response_

A physical model is proposed in which the meridians assist in the ion drift based on statistics. The direction of the meridian can be considered as a built-in force that assists the movement of ions. The high velocity of the ions indicates that the ions drift from one acupoint to another in a short time. According to the current density formula [26], the short arrival time of ions between two acupoints leads to an increase in the *I*_response_. For example, the direction of the Sanyang meridian of the hand and the inputted electric field are the same, and positive ions drift because of two forces. Thus, a short *τ*_rising_ and a high *I*_*R,S*_ are observed. Figure 10 also shows the schematic of the flow direction of the meridian, the direction of *I*_drift_, and the *I*_response_ relationship.

The results illustrate that the meridian direction can be measured and prove that the meridian direction is identical to that inferred in this work and mentioned in TCM. Notably, the findings of this study can be applied to clinical practices. When there is chest/heart discomfort, acupuncture over the Taiyin lung meridian of the hand and electric acupuncture from FHP to NHP can be applied. However, the association between the clinical therapeutic effect and meridian direction requires further research.

## Conclusion

In this study, we analyzed the response current, which relies on the input AC voltage on the meridians. Based on the parameters, *τ*_rising_ and *I*_R,S_, relating to the response current of the meridians, the model of meridians assisting in ionic drift was proposed, and the meridian direction was also inferred. The result showed that the direction of the meridian obtained in this work was consistent with those in TCM. This study affords more analytical methods for use in clinical practices.

## Data Availability

All data are fully available without restriction.

## Abbreviations

TCM: Traditional Chinese medicine
WHO: World Health Organization
MEAD: Meridian energy analysis device
I–t: Current–time
ITIC: Isothermal transient ionic current
ITP: Idiopathic thrombocytopenic purpura
LU: *Taiyin* lung meridian in hand
PC: *Jueyin* pericardium meridian in hand
HT: *Shaoyin* heart meridian in hand
ST: *Yangming* stomach meridian in foot
GB: *Shaoyang* gallbladder meridian in foot
BL: *Taiyang* bladder meridian in foot
AC: Alternating current
NHP: Near heat point
FHP: Far heat point
*I*_response_: Response current
*I*_drift_: Drift current
*V*_Low_: Negative voltage
*V*_High_: Positive voltage
*I*_*R*__,__*S*_: Saturated I_response_
